# Characterization of Highly Mucus-Adherent Non-GMO Derivatives of *Lacticaseibacillus rhamnosus* GG

**DOI:** 10.3389/fbioe.2020.01024

**Published:** 2020-08-19

**Authors:** Pia Rasinkangas, Hanne L. P. Tytgat, Jarmo Ritari, Justus Reunanen, Seppo Salminen, Airi Palva, François P. Douillard, Willem M. de Vos

**Affiliations:** ^1^Department of Veterinary Biosciences, Faculty of Veterinary Medicine, University of Helsinki, Helsinki, Finland; ^2^Laboratory of Microbiology, Wageningen University, Wageningen, Netherlands; ^3^Functional Foods Forum, Faculty of Medicine, University of Turku, Turku, Finland; ^4^Human Microbiome Research Program, Faculty of Medicine, University of Helsinki, Helsinki, Finland

**Keywords:** pili, mucus, *Lacticaseibacillus rhamnosus* GG, adhesion, probiotics, non-GMO

## Abstract

*Lacticaseibacillus rhamnosus* GG is one of the best studied lactic acid bacteria in the context of probiotic effects. *L. rhamnosus* GG has been shown to prevent diarrhea in children and adults and has been implicated to have mitigating or preventive effects in several disorders connected to microbiota dysbiosis. The probiotic effects are largely attributed to its adhesive heterotrimeric sortase-dependent pili, encoded by the *spaCBA-srtC1* gene cluster. Indeed, the strain-specific SpaCBA pili have been shown to contribute to adherence, biofilm formation and host signaling. In this work we set out to generate non-GMO derivatives of *L. rhamnosus* GG that adhere stronger to mucus compared to the wild-type strain using chemical mutagenesis. We selected 13 derivatives that showed an increased mucus-adherent phenotype. Deep shotgun resequencing of the strains enabled division of the strains into three classes, two of which revealed SNPs (single nucleotide polymorphisms) in the *spaA* and *spaC* genes encoding the shaft and tip adhesive pilins, respectively. Strikingly, the other class derivatives demonstrated less clear genotype – phenotype relationships, illustrating that pili biogenesis and structure is also affected by other processes. Further characterization of the different classes of derivatives was performed by PacBio SMRT sequencing and RNAseq analysis, which resulted in the identification of molecular candidates driving pilin biosynthesis and functionality. In conclusion, we report on the generation and characterization of three classes of strongly adherent *L. rhamnosus* GG derivatives that show an increase in adhesion to mucus. These are of special interest as they provide a window on processes and genes driving piliation and its control in *L. rhamnosus* GG and offer a variety of non-GMO derivatives of this key probiotic strain that are applicable in food products.

## Introduction

The last decade of microbiome analysis has rendered a plethora of information on the bacteria inhabiting a healthy or diseased microbiota-gut ecosystem. Dysbiosis of the gut microbiota has been correlated to a large variety of non-communicable diseases, like diabetes, obesity and even autism (Qin et al., 2010). Building further on this knowledge, it is key to support these correlations with hard-core microbiology insights: understanding how bacteria can mediate and influence human physiology. These insights also pave the way for novel application opportunities, for instance by understanding how microbiota composition and crosstalk can be modulated. One possibility is the use of beneficial microbes or probiotics, defined as live microorganisms, that, when administered in adequate amounts, confer a health benefit to the host (FAO/WHO, 2001).

Many of the most widely studied probiotics are lactic acid bacteria, with *Lacticaseibacillus rhamnosus* GG being a prime example (de Vos, 2011). This strain was previously known as *Lactobacillus rhamnosus* GG (Zheng et al., 2020). Its probiotic potential ranges from the prevention of antibiotic-associated diarrhea in children and adults (Arvola et al., 1999; Szajewska and Kolodziej, 2015), over atopic diseases (Kalliomaki et al., 2001; Nermes et al., 2011), to respiratory tract infections in children (Hatakka et al., 2001; Kumpu et al., 2012; Luoto et al., 2014). As also demanded by EFSA (European Food Safety Authority), health claims should be corroborated by defined studies of probiotic effectors (Binnendijk and Rijkers, 2013).

One of the key features of *L. rhamnosus* GG are its highly adherent, long (1 or more microns) SpaCBA-pili (Kankainen et al., 2009) that play a key role in adherence, host signaling, biofilm formation and competitive exclusion of pathogens like *Enterococcus faecium* (Lebeer et al., 2012; Ardita et al., 2014; Tytgat et al., 2016a, b; Chandrasekharan et al., 2019). These sortase-dependent pili are heterotrimers of a major, shaft-forming pilin SpaA, a SpaB polymerization termination signaling pilin and a pilus-decorating SpaC pilin (Reunanen et al., 2012; Douillard et al., 2014). It has been shown that these species-specific pili, encoded in a *spaCBA-srtC1* cluster, are the main drivers of the high colonization phenotype of *L. rhamnosus* GG and are for instance absent in the closely related Lc705 strain (Kankainen et al., 2009; von Ossowski et al., 2010; Lebeer et al., 2012; Ardita et al., 2014). Especially the SpaC pilin was found to be crucial, as it is the major contributor to mucus binding of *L. rhamnosus* GG, probably via its C-terminal collagen-binding and N-terminal von Willebrand factor domains (Tripathi et al., 2013; Kant et al., 2016). The three pilin subunits are assembled into the mature SpaCBA pili by a pilin-specific transpeptidase, SrtC1, which is encoded in the same gene cluster as the three pilins, whilst a housekeeping sortase, SrtA, assures attachment to the cell wall (Hendrickx et al., 2011; Reunanen et al., 2012). The mechanisms and motifs by which these sortases act, have been studied in-depth in *L. rhamnosus* GG (Douillard et al., 2014, 2016). This has enhanced the general knowledge on pilus assembly and function of these key cell wall appendages of a multitude of Gram-positive species, including pathogens (Mandlik et al., 2008).

To further expand our insights in the structure and function of the SpaCBA pili, we use random chemical mutagenesis to generate non-GMO derivatives (The European Union, 2001) of *L. rhamnosus* GG. This approach has a three-fold goal: (1) the generation, screening and characterization of strains with an aberrant pilosotype, (2) the generation of strains safe to use in human trials with potential enlarged beneficial properties, and (3) advancing our understanding of the processes and genes driving piliation and its control. We already successfully used this non-GMO approach to produce and describe non-piliated variants of *L. rhamnosus* GG (Rasinkangas et al., 2014).

In this work we focus on the generation of derivatives of *L. rhamnosus* GG showing an increased capacity to adhere to mucus, using a similar approach: random chemical mutagenesis followed by genome resequencing. In what follows we describe the generation, selection and isolation of 13 highly mucus-adherent *L. rhamnosus* GG derivatives, followed by their phenotypic, biochemical, genomic and transcriptomic characterization.

## Materials and Methods

### Bacterial Strains and Growth Conditions

*Lacticaseibacillus rhamnosus* GG (ATCC53103) was obtained from Valio Ltd., Helsinki, Finland. As a pilus-less control, the previously characterized *L. rhamnosus* GG PB12 strain was included (Rasinkangas et al., 2014). Strains were grown in De Man-Rogosa-Sharpe (MRS) medium and 1.5% agar (Difco) at 37°C.

### EMS Mutagenesis

Ethyl methanesulphonate (EMS, Sigma Aldrich) was used as an alkylating mutagen (Sega, 1984; Parekh et al., 2000), as described in our previous work on the production of non-piliated derivatives of *L. rhamnosus* GG (Rasinkangas et al., 2014). In particular, stationary-phase *L. rhamnosus* GG cells were treated with 2% (v/v) EMS and incubated at 37°C for 2 h. After incubation, samples were washed several times with phosphate-buffered saline (PBS, pH 7.3, Oxoid) and resuspended in MRS broth. Bacterial survival was estimated by plating a dilution series of the samples at 16%. At various stages in the mutation selection process, cultures were stored at −80°C, after supplementation with 20% glycerol.

### Enrichment of Highly Piliated Strains and High-Throughput Dot Blot Screening of Derivatives

A screening was set up for derivatives of *L. rhamnosus* GG that could present a stronger interaction with mucus. Given that the SpaC pilin is the main mucus-interacting component of the SpaCBA-pili, and by extension of *L. rhamnosus* GG, an anti-SpaC antibody based screen was set up (Kankainen et al., 2009; von Ossowski et al., 2010; Lebeer et al., 2012; Nishiyama et al., 2016). A dense EMS-treated bacterial suspension of OD_600 *nm*_ 4 was incubated in wells coated with anti-SpaC IgG, purified from anti-SpaC rabbit antiserum (Kankainen et al., 2009). After 3–4 h incubation, wells were washed ten times with PBS, followed by scraping to release potentially highly adherent *L. rhamnosus* GG derivatives. These scraped cells were incubated overnight in MRS medium. This enrichment scheme was repeated a total of three times. Following the last round of enrichment, a dilution series was prepared and plated. Colonies were picked, grown and screened using a dot blot assay as described earlier (Rasinkangas et al., 2014). The strains exhibiting the strongest interaction with anti-SpaC IgG in the dot blot screening were selected for further characterization.

### Illumina-Based Genome Screening of Derivatives

Genomic DNA was extracted using the Wizard Genomic DNA purification kit (Promega). Genomes were sequenced in paired-end mode using the MiSeq Illumina platform. MUMmer 3.0 software (Kurtz et al., 2004) was used to align sequence assemblies to the *L. rhamnosus* GG genome (Kankainen et al., 2009). MIRA software was then used to align sequences and annotate mutations (Chevreux et al., 1999). Illumina paired end Fastq reads were mapped against *L. rhamnosus* GG (FM179322.1) annotated GenBank file using MIRA v4.0.2 with the following parameters: “job = mapping,genome,accurate,” “parameters = COMMON_SETTINGS -NW:cac = no,” “technology = solexa” and “autopairing.” Commands “miraconvert -t asnp” and “miraconvert -t hsnp” were used to generate single nucleotide polymorphisms (SNP) analysis results. Mutations residing in the *spaCBA-srtC1* gene cluster were further verified with Sanger sequencing.

### PacBio Next-Generation Sequencing of Class Representatives

Genomic DNA extraction was performed as described above. Further processing, sequencing and variant detection (SNP) in representative strains (PS17, PS24, PS31, and PS41) of each class was performed by BaseClear (Leiden, Netherlands) according to their procedures (paired-end sequencing Illumina HiSeq2500). In the case of PS24 and PS31, representatives of Class II and III, de novo hybrid genome assemblies were performed by BaseClear and compared to the genome of wild-type *L. rhamnosus* GG to detect any large aberrations in the genome structure of these derivatives (PacBio Sequel Instrument, gapped regions were closed with Illumina reads).

### Qualitative and Quantitative Analysis of Surface-Located Pilins Using Immunofluorescence

Conforming to our earlier studies, we performed immunofluorescence labeling, quantification and microscopy of pilins on the cell surface of the selected derivatives (Rasinkangas et al., 2014). SpaA and SpaC antisera were used as primary labeling agent, followed by detection via secondary labeling with Alexa Fluor 488-labeled goat anti-rabbit antibody (Invitrogen). DAPI (1:1000 dilution, Dilactate form, Thermo Fisher Scientific) was used to stain and thus quantify all cells present in a sample, thus enabling normalization of the fluorescence intensity results for SpaA and SpaC. Fluorescence intensities of each stain were measured separately using a Victor3 1460 multilabel counter (Perkin Elmer). Normalization was performed by dividing the obtained fluorescence intensity values by the corresponding DAPI intensity for each strain. Both quantitative and qualitative data from a representative experiment is shown.

### Immuno-Electron Microscopy of SpaA and SpaC Pilins

Experimental details are in accordance with (Douillard et al., 2014; Rasinkangas et al., 2014). In short, stationary grown cells were washed with PBS and incubated for 1 h on top of Formvar-carbon-coated grids. Grids were subsequently washed several times with 0.02 M Glycine in PBS and blocked using 1% BSA in PBS for 15 min. After blocking, grids were incubated with antiserum diluted 1:100 in blocking solution for 1 h. A protein A-gold suspension either containing 5 or 10 nm gold particles was diluted 1:55 and incubated with the grids for 20 min after several washes with 0.1% BSA in PBS. SpaA and SpaC pilins were detected by coupling SpaA antiserum to 10 nm and SpaC to 5 nM protein A-gold particles. Grids were fixed with 1% (v/v) gluteraldehyde (Sigma-Aldrich) and washed with water, followed by staining them using a 1.8% methylcellulose-0.4% uranyl acetate mixture on ice. Samples were visualized using a JEM-1400 transmission electron microscope (JEOL).

### Western Blot Analysis of SpaC and SpaA Pilins

Cell wall proteins were extracted from cultures of which the OD_600 *nm*_ was adjusted to 8 prior to extraction, as described in Rasinkangas et al. (2014). Protein extracts were diluted 1:300 and 1:200 respectively for detection using SpaA and SpaC antisera on Western blot. To visualize the total quantity of proteins in the blots, a Western blot of 1:200 diluted samples (similar to SpaC blot) was developed using a whole cell antibody (WCA, 1:25000 dilution) against *L. rhamnosus* GG (Tytgat et al., 2016b).

### Mucus Adherence Measurements

Strains were grown overnight in the presence of ^3^H-labeled thymidine (Perkin Elmer). Cultures were then incubated in a Maxisorp 96-well plate coated with either porcine type II mucus (Sigma-Aldrich) or human mucus, followed by extensive washing with PBS (Oxoid) and lysis of the remaining adherent cells. Sample radioactivity was measured using a Wallac 1480 Wizard 3 automatic gamma counter (Perkin Elmer). Full experimental details are described in Vesterlund et al. (2005). Each assay was performed in three biological repeats, with each a minimum of three technical replicates per strain.

### Transcriptomic Analysis of *L. rhamnosus* GG Derivatives

Wild-type *L. rhamnosus* GG, PS24 and PB12 as negative control were grown in MRS till OD_600 *nm*_ 2 (early exponential) and OD_600 *nm*_ 7 (late exponential, early stationary phase). Cell pellets were resuspended in TRIzol (Life technologies) and RNA extraction was performed with the RNeasy kit (Qiagen) according to manufacturers’ instructions (triplicate for wild-type). Quality and quantity of RNA was monitored using Qubit (Thermo Fisher) and Experion (Bio-Rad). rRNA was depleted using the RiboZero kit (Epicentre, Ilumina), followed by cDNA library preparation according to the guidelines of the ScriptSeq v2 RNA-Seq Library Preparation Kit (Epicentre, Illumina).

HighPrep PCR magnetic beads (Magbio) enabled cDNA purification prior to barcoding with ScriptSeq Index PCR Primers (Epicentre, Illumina). The RNA-Seq library was again further purified using HighPrep PCR magnetic beads (Magbio) and quantity and quality was assessed using Nanodrop (Thermo Fisher Scientific). Barcoded cDNA libraries were pooled and sent to Baseclear (Leiden, Netherlands) for sequencing on one single lane using the Illumina HiSeq2500 platform. All bioinformatic work was performed on a local Galaxy webserver (Giardine et al., 2005; Blankenberg et al., 2010; Goecks et al., 2010). rRNA reads were removed with SortMeRNA version 2.0 (Kopylova et al., 2012) and all included databases. Trueseq adapters were trimmed with Cutadapt v1.8.1 (Martin, 2011) using default settings. Quality trimming was performed with Sickle version 1.33 (Joshi and Fass, 2011) with a minimum sequence length of 50 bp and a minimum quality threshold of 30. Reads were mapped to the genome with Bowtie2 version 2.2.4 (Langmead and Salzberg, 2012) using default settings and mapping rate was inspected with Samtools version 0.1.19 (Li et al., 2009). Gene coverage was calculated with R version 3.1.2 (R Core Team, 2014) and the R package HTSeq-count version 0.6.1 (Anders et al., 2015). GFF files were obtained by converting Genbank files via Readseq version 2.1.19 (Gilbert, 2003). Differential expression was calculated with the R package DESeq2 version 2.1.8.0 (Love et al., 2014).

### Sequence Accession Numbers

The genome sequences and RNASeq data of the described derivatives can be found in the NCBI Sequence Read Archive (SRA) database under BioProject accession code PRJNA309744. Specific codes of the highly mucus-adherent strains are:

PS11 – SAMN04440341;

PS12 – SAMN04440342;

PS13 – SAMN04440343;

PS14 – SAMN04440344;

PS15 – SAMN04440345;

PS16 – SAMN04440346;

PS17 – SAMN04440347;

PS21 – SAMN04440348;

PS22 – SAMN04440349;

PS23 – SAMN04440350;

PS24 – SAMN04440351;

PS31 – SAMN04440352;

PS41 – SAMN04440353.

### Data Analysis

All analyses were performed using GraphPad Prism 8. Significant differences between two groups were calculated using unpaired *t*-tests and the significance level was set at *p* < 0.05.

## Results

### Selection of Highly Mucus-Adherent *L. rhamnosus* GG Derivatives

The aim of the present study was to decipher whether it would be possible to produce highly mucus-adherent derivatives of *L. rhamnosus* GG using random mutagenesis induced by the chemical mutagen ethyl methanesulfonate. Benefits of these derivatives include that they are considered to be non-GMO (The European Union, 2001) and that they can offer unique insights in the genes driving *L. rhamnosus* GG piliation.

By coating a plate with anti-SpaC IgG we aimed to enrich derivatives that presented a higher fraction of SpaC pilins, the major adhesive pilin of the *L. rhamnosus* GG SpaCBA pili, on their cell surface. A total of 384 strains that matched this phenotype was screened, to ultimately obtain 13 strains, which, according to dot blot analysis, produced higher quantities of SpaC compared to the parental wild-type *L. rhamnosus* GG strain (results not shown). This results in a discovery rate of 3.4%, which is in line with our earlier work on the production of pilus-less derivatives (Rasinkangas et al., 2014). In what follows we characterize these 13 “PS” strains in-depth by focusing on their genotypes and phenotypes.

### Genomic Resequencing of the Selected Derivatives

The genotype of the 13 selected strains was analyzed by shotgun genomic resequencing using Illumina technology to identify SNPs (Singe Nucleotide Polymorphisms) (Table 1 for a summary, Supplementary File S1 for the detailed results of all selected strains). Based on the mutation profile that emerged, the 13 strains were divided into three classes (Table 1): Class I consists of 7 strains denoted as PS11–PS17, Class II of 5 strains termed PS21-PS24 and PS31, and Class III of the single strain, PS41, respectively. A total of 7 mutations, localized both in coding sequences (3) and intergenic regions (4) were found to be shared amongst all derivatives (Supplementary Table S1).

**TABLE 1:** Mutations detected by Illumina resequencing in the selected highly adherent derivatives of *L. rhamnosus* GG.

		Phenotype driving mutation			Other mutations
		
Strain	Class	Gene	Mutation	AA change	SNP#
PS11	I	*spaC*	C → T	Pro552Ser	46
PS12			C → T	Pro552Ser	58
PS13			C → T	Pro552Ser	59
PS14			C → T	Pro552Ser	47
PS15			C → T	Pro552Ser	62
PS16			C → T	Pro552Ser	54
PS17			C → T	Pro552Ser	51
PS21	II	Unknown	–	–	41
PS22			–	–	65
PS23			–	–	73
PS24			–	–	44
PS31			–	–	80
PS41	III	*spaA*	C → T	Thr35Met	35

*SNP#: Quantity of other single nucleotide mutations in the strain.*

Class I strains PS11–PS17 were clustered based on the emergence of the same mutation in the gene encoding SpaC (Table 1). The mutation resulted in an amino acid change (Pro → Ser) of residue 552 of the SpaC protein, which is located at the C-terminal end of the collagen-binding domain of SpaC (Tripathi et al., 2013; Kant et al., 2016). Apart from this SNP in the *spaCBA-srtC1cluster*, there are 38 SNPs shared by all Class I mutants (see Supplementary Table S1).

Genomic analysis of derivatives belonging to Class II (PS21–PS24, PS31) did not result in a clear genotype (Table 1). Based on the shotgun sequencing, no mutations in genes that were in a direct way related to piliation could be detected.

The strain constituting Class IV, PS41, presented a mutation in the gene encoding SpaA. The mutation resulted in a Thr → Met substitution in residue 35, which is close to the predicted secretion signal peptide cleavage site (residues 1–33) of this pilin (Table 1).

### Quantification of Pilins Using Immuno-Fluorescence Labeling

**FIGURE 1:**
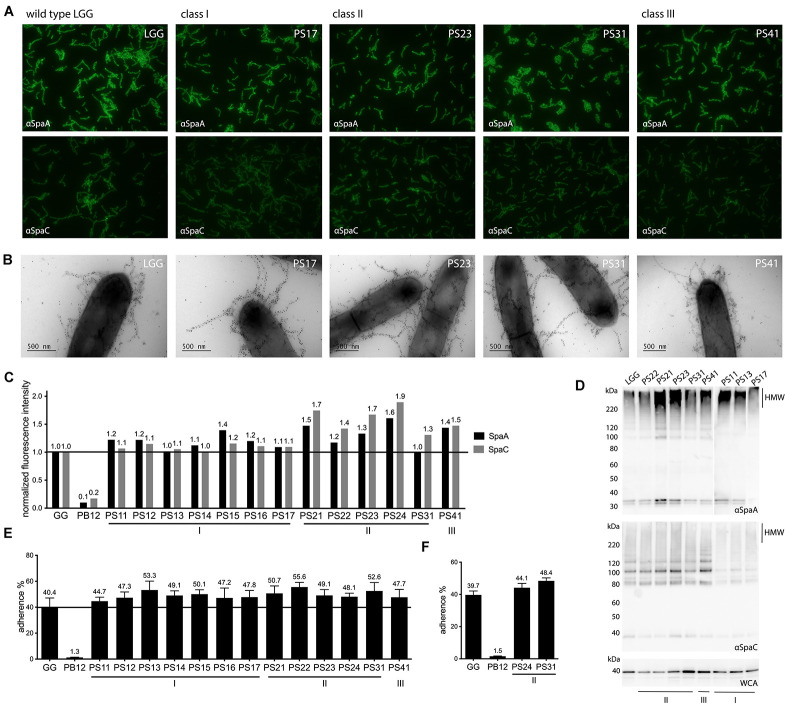
Phenotypic assessment of piliation and mucus adhesion of highly mucus-adherent derivatives of *L. rhamnosus* GG. **(A)** Immunofluorescent labeling of SpaA and SpaC on cell surface of derivatives. Strains were labeled with either SpaA or SpaC antisera and detected with Alexa 488-labeled secondary antibody. Representative images are included for each strain. **(B)** Immunoelectron micrographs of strains double labeled with SpaC and SpaA antibodies. Both SpaC (5 nm gold particles) and SpaA (10 nm gold particles) are imaged on the pili of the different derivative strains (a representative figure is shown for each strain). **(C)** Quantitative immunofluorescent labeling results of SpaA and SpaC. Fluorescent signals of strains labeled with either SpaA and SpaC (detected by Alexa 488-labeled antibodies) were normalized to DAPI fluorescence. These values were further normalized to the fluorescence of the parental *L. rhamnosus* GG strain (indicated with a line to facilitate interpretation). A representative experiment is depicted. **(D)** Western blot analysis of cell wall associated proteome of each derivative. The pilus ladder (HMW for High Molecular Weight fraction) of the derivatives was visualized by probing cell wall extracts of each strain with SpaA and SpaC antibodies. Total amount of proteins is shown as a reference with a whole cell antibody (WCA) blot. **(E)** Mucus adhesion of the derivatives. The average result of 3 biological measurements (with each 3–6 technical repeats) is depicted for each derivative with wild-type *L. rhamnosus* GG as a positive control and the non-piliated derivative PB12 as a negative control. Standard error of mean (SEM) is shown. **(F)** Adhesion to human mucus. Adhesion of two derivatives, together with the positive and negative controls, to mucus isolated out of a human specimen, to validate porcine results. The average of three biological repeats is depicted with standard error of mean (SEM).

To be able to evaluate whether the strains produce higher quantities of pili compared to the parental strain *L. rhamnosus* GG, both SpaA and SpaC pilins of all derivatives were both qualitatively and quantitatively assessed in immuno-fluorescence labeling assays (Figure 1A). Quantitative measurements were obtained by normalizing fluorescence to DAPI staining of nucleic acids, and ultimately to values of the wild-type strain.

Pilin quantities were highly similar for all derivatives of Class I, and slightly higher for the SpaA pilin (1.0–1.4 for SpaA and 1.0–1.1 for SpaC) compared to wild-type (SpaA and SpaC normalized to 1.0). For the two other classes, a slight increase in pilin abundance, especially of SpaC pilin quantities, could be recorded (Figure 1C). Indeed, the strains of Class II all had an increased abundance of SpaC (1.3–1.9), compared to SpaA. Interestingly, in Class III the increase in abundance of SpaA and SpaC pilins seems to be balanced, both showing a considerable (almost 50%) increase in abundance compared to the parental strain (resp. 1.4 and 1.5).

These results indicate that the mutation in the *spaC* gene in the Class I mutants seems to slightly affect the abundance of SpaC in the pili, without affecting overall pili quantity. Classes II and III, in contrast, do show an increase of both SpaA and SpaC pilins, which might point toward a higher degree of piliation of these strains.

### Analysis of the Architecture and Size of the Pili in the Different Derivative Classes Using Immuno-Electron Microscopy and Western Blot Analysis

Since immuno-fluorescence does not provide information about the architecture and size of the pili, we used immuno-electron microcopy (EM) to visualize the pili in the different classes of the 13 highly mucus adherent strains (Figure 1B). In general, the pili produced by the strains seemed to be similar in size to the parental strain *L. rhamnosus* GG, and no apparent large differences in pili morphology could be observed.

To investigate potential differences in the pilus ladder (Kankainen et al., 2009), cell wall-associated pilins from all strains were analyzed by Western blot and probed with anti-SpaC and -SpaA antibody (Figure 1D). These analyses revealed that the pilus ladder was highly similar for all strains and did not reveal apparent aberrations in the pilin ladders of PS strains compared to each other or the parental strain, *L. rhamnosus* GG.

### Mucus Adhesion of the Selected Derivatives

As the SpaCBA pili of *L. rhamnosus* GG play a key role in its strong adhesive phenotype (Kankainen et al., 2009), we tested how the altered piliation of the different derivative classes affected mucus adhesion. Binding to porcine mucus, as a proxy for human mucus, was evaluated for all strains.

Wild-type *L. rhamnosus* GG presented an adhesion percentage of 40.4% (standard error of mean, SEM 6.7%) (Figure 1E). The earlier-characterized pilus-less derivative PB12 was used as a negative control and exhibited a statistically significant lower adhesion to mucus compared to wild-type and all derivatives (1.3%, SEM 0.2%, *p* < 0.05) (Rasinkangas et al., 2014). All derivative strains showed an increase in mucus adhesion with percentages ranging between 44.7 and 55.6% of cells irreversibly bound to mucus (Figure 1E). Strikingly, no clear differences or trends could be discerned between the different derivatives or classes.

Since porcine mucus was used for this analysis, two derivatives Class II (resp. PS24 and PS31) were retested on human mucus in order to evaluate if results were comparable between the two mucus sources (Figure 1F). Indeed, both strains showed a higher adherence to human mucus (44.1 and 48.4% resp.) compared to wild-type *L. rhamnosus* GG (39.7%) and certainly compared to the negative control strain, the pilus-less PB12 derivative (1.5%, *p* < 0.05) (Figure 1F).

### Genomic Characterization of Derivatives by Single-Molecule Sequencing

For both Class I and Class III derivatives, shotgun (Illumina) sequencing could identify a mutation respectively in the adhesive pilin SpaC or in the major pilin SpaA. However, for the Class II derivatives the driving mutation affecting a higher SpaC antibody interaction remained elusive. Therefore, we performed single-molecule (PacBio) analysis of a representative of each class to confirm the genotype uncovered by the shotgun sequencing (Supplementary File S2). All classes had several SNPs in a pseudogene in common, *LGG_RS15025*.

The single-molecule analysis confirmed the genotype of the Class I representative PS17, namely a SNP in the adhesive pilin subunit SpaC, and for Class III strain PS41, a SNP in the major pilin SpaA (Supplementary File S2). Of particular interest was the analysis of two representative strains of Class II, PS24 and PS31 respectively. Whole genome alignment of both PS strains did not reveal gaps, large indels, inversions or other apparent anomalies compared to wild-type *L. rhamnosus* GG (Supplementary File S3).

The single-molecule analysis of PS24 resulted in an updated list of 43 genes affected by single nucleotide variations (Supplementary File S2). None of the SNPs was located in the *spaCBA-srtC1* gene cluster, confirming the shotgun results (Supplementary File S1). Also in accordance with the shotgun sequencing results, a SNP was detected in the SpaD pilus protein (Asp → Asn). SpaD is the major pilin of a second pili cluster (SpaFED) present in *L. rhamnosus* GG, which is thought to only play a minor role in interaction with the environment compared to the SpaCBA pili cluster, as only the latter seem to be expressed by the bacterium (von Ossowski et al., 2010; Reunanen et al., 2012). Of note, another LPXTG-motif harboring adhesin encoded by *LGG_RS13990* (*LGG_02923*) also harbors a SNP.

Further striking observations include the high amount of mutations in genes potentially affecting pilin production, like the gluconate operon repressor family regulator *farR* (*LGG_RS13185, LGG_02757*), transporters, surface and membrane proteins. Especially the large amount metal and cation transporters affected by SNPs stand out: *LGG_RS11535* (*LGG_02411, mntH*), *LGG_RS11610* (*LGG_02426, psaA*), *LGG_RS12705* (*LGG_02658*), and *LGG_RS11565* (*LGG_02417*).

In the Class II derivative PS31, a total of 79 genes were affected by SNPs. It is hard to discern any large trends in the results, but one of the potential effector mutations could be in the gene of a transcriptional regulator belonging to the MarR family (*LGG_RS10240*, *LGG_02127*).

### Transcriptome Analysis of the Class II Derivative PS24

To further investigate the different leads resulting from the SNP analysis of the Class II derivative PS24, the transcriptome of the strain was investigated at early (OD_600__*nm*_ 2) and late exponential phase (OD_600__*nm*_ 7) and compared to wild-type *L. rhamnosus* GG and the pili-less PB12 derivative (Rasinkangas et al., 2014).

RNA was isolated from wild-type *L. rhamnosus* GG, PB12 and PS24 and analyzed by RNASeq using deep Illumina sequencing. All resulting count tables and differential expression analysis comparing expression between *L. rhamnosus* GG and the PS24 derivative are compiled in Supplementary File S4.

**FIGURE 2:**
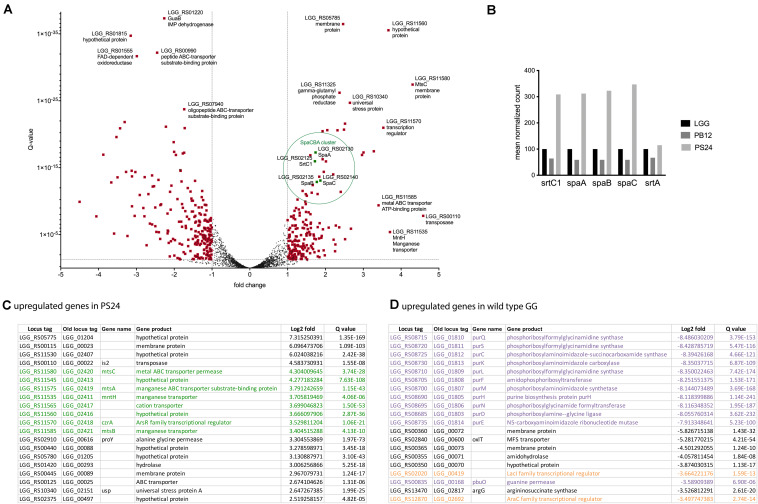
Transcriptomic analysis of PS24 compared to wild-type *L. rhamnosus* GG at OD_600 *nm*_ 2. **(A)** Volcano plot of differential expression compared for *L. rhamnosus* GG and PS24. Log2 fold change versus *q*-value is depicted. Data points marked in red have a log2 fold larger than 1 and a *q*-value smaller than 0.05. Positive fold change implies higher expression in the PS24 strain. **(B)** Mean normalized pili transcript count. Normalized expression of the *spaCBA-srtC1*gene cluster in the pilus-less PB12 derivative and PS24 derivative relative to wild-type *L. rhamnosus* GG. The housekeeping sortase A was included for comparison. **(C)** Top 20 upregulated genes in PS24 derivative. The top 20 genes with the highest log2fold value compared to wild-type *L. rhamnosus* GG at OD_600 *nm*_ 2. **(D)** Top 20 downregulated genes in PS24 derivative. The top 20 genes with the highest log2fold value in wild-type *L. rhamnosus* GG at OD_600 *nm*_ 2, compared to the PS24 derivative.

In line with our observations described above and the goal of the experiment, the genes of the *spaCBA-srtC1* cluster are among the genes that are strongly upregulated in the PS24 derivative compared to the parental strain in early exponential phase (Figure 2A). Transcription levels of the genes of the other pili cluster, *spaFED-srtC2*, in contrast were extremely low in all strains and conditions tested (Supplementary File S4), confirming earlier observations stating that the SpaFED-pili do not play a role in the strong adhesion capacity of *L. rhamnosus* GG strains. Further comparison of normalized counts for the *spaCBA-srtC1* cluster for wild-type, the pilus-less PB12 and the PS24 strain resulted in a strong increase in expression of the *SpaCBA-srtC1* gene cluster in the PS24 strain (Figure 2B). This trend is both apparent at the early (OD_600 *nm*_ 2) (Figure 2B) and late exponential growth phase (OD_600 *nm*_ 7) time points (see Supplementary File S4).

Analysis of the 20 most upregulated genes in PS24, compared to wild-type, further corroborate the hypothesis that cation homeostasis might play a regulatory role affecting piliation and mucus adhesion in *L. rhamnosus* GG. Approximately 40% of the top hits belong to a cluster of genes related to cation transport (Figures 2A,C, green). Two genes in particular stand out, as they are harboring a SNP in the PS24 strain and both encode for a cation transporter, namely *LGG_RS11535* (*LGG_02411, mntH*) and *LGG_RS11565* (*LGG_02417*) (Supplementary File S3).

Analysis of the counterpart, the 20 most downregulated genes in PS24 compared to the parental strain (Figure 2D), showed a strong downregulation of the C1 metabolism in PS24, more in particular genes involved in purine metabolism (purple in Figure 2D). Two genes that are also strongly downregulated in PS24, include two transcriptional regulators (orange in Figure 2D).

## Discussion

In this study, we sought to find highly mucus adherent derivatives of *L. rhamnosus* GG with the means of random chemical mutagenesis. Such derivatives not only can render invaluable insights in processes steering pili biogenesis and functionality, but also can safely be used in a human trial setting as these derivatives are considered as non-GMO (The European Union, 2001).

Increased binding to anti-SpaC IgG was chosen as a readout to select strains, as SpaC is the pilin mainly responsible for the strong adhesive properties attributed to the SpaCBA pili of *L. rhamnosus* GG (Kankainen et al., 2009; von Ossowski et al., 2010; Lebeer et al., 2012; Reunanen et al., 2012; Tripathi et al., 2013). Combined mutagenesis and selection led to the isolation of 13 highly mucus-adherent strains. Illumina genomic re-sequencing revealed mutations underlying the observed phenotype and allowed classification of the derivatives into 3 classes. The strains were also subjected to further phenotypic analyses to assess how piliation was affected in the different classes.

All selected strains showed an increased capability to adhere to mucus compared to wild-type *L. rhamnosus* GG (Figure 1D). This confirms the validity of our screening set-up, where interaction with anti-SpaC IgG is used as a predictor of mucus adhesion capacity. These findings also corroborate the key role of SpaC in the strong mucus adhesion phenotype of *L. rhamnosus* GG. Our results also further validate the use of porcine mucus as a proxy for human mucus as the heightened mucus adhesion phenotype could be replicated for two selected strains (Figure 1F).

With mucus adherence of wild-type *L. rhamnosus* GG being around 40% both for porcine and human mucus, a 15% increase in adhesion (PS22, approximately 55%) was the strongest increase in mucus adhesion that could be registered for the derivatives. This is a further attestation of the known superb adhesion capacity of wild-type *L. rhamnosus* GG (Kankainen et al., 2009; von Ossowski et al., 2010; Reunanen et al., 2012; Tytgat et al., 2016a) and suggesting that this is highly optimized through evolution as only relatively modest increases in mucus adhesion could be achieved.

Class I mutants were found to harbor a mutation in the C-terminal of the *spaC* gene, close to its collagen-binding domain. These strains, however, did not appear to produce more pili in general or represent more SpaC on their cell surface (Figure 1). The higher adhesion efficacy of Class I strains is likely due to a direct impact of the mutation on the adherence properties of the SpaC tip pilin itself, potentially by altering its collagen-binding domain. Further studies are needed to understand the exact mechanisms driving SpaC adherence and the role of its different domains. Interestingly, all derivatives of Class I also share SNPs in other genes and intergenic regions. Further research is necessary to assess the importance, if any, of these SNPs, by for instance reverse engineering.

The strain designated as a Class III strain, PS41, harbors a mutation in the gene encoding SpaA, the shaft pilin, which is located close to its secretion signal. This mutation may affect the secretion efficiency of backbone pilin SpaA and hence result in higher pilus production. Another potential explanation is the production of longer pili. In *Corynebacterium diphtheriae*, pilus length is shown to be regulated by the ratio of major pilin SpaA to basal pilin SpaB, and a higher quantity of SpaA tends to lead to formation of longer pili (Swierczynski and Ton-That, 2006). Our selection of the PS41 strain based on strong interaction with anti-SpaC IgG might thus be due either to a higher presentation of SpaC along the pilus, or on more pili, or more accessible SpaC units on longer pilins. PS41 was found to produce a higher amount of both SpaA and SpaC pilins compared to the parental strain (Figure 1C). This, however, did not result in a significant increase in the length of the pili or changes in the pilin protein ladder (Figures 1B,D). Our results do point toward a higher degree of piliation in general on the surface of PS41, which is likely coming from a higher secretion of SpaA (Figures 1A,B). This is corroborated by the balanced increase in relative abundance of SpaA and SpaC subunits in PS41 compared to wild-type and the other derivatives.

In contrast to Class I and III, where a SNP was found that could be directly linked to genes driving SpaCBA biogenesis, the genotype of the Class II strains remains more elusive, nevertheless this class harbors the strains that demonstrate the most dramatic increase in the amount of SpaCBA pili (Figure 1). Considering the phenotype, i.e., high mucus adherence and high production of pilins, it is likely that mutations lead to more efficient gene transcription or increased secretion of the pilins by an unknown mechanism. However, at this point no mutations that are known to influence pilin biogenesis or functionality could be identified in genomic analyses of strains this class. As the secretion machinery of *L. rhamnosus* GG is still largely unknown, it is difficult to evaluate which of the genes would impact the quantity of the pilins that are secreted and competent for pili production. Single-molecule re-sequencing of two representatives of this class (PS24 and PS31) showed that no large indels, inversions or other genomic events could explain the observed phenotype (Supplementary File S3).

Dedicated PacBio SNP analysis of Class II mutant PS24 revealed mutations in the *spaFED-srtC2* pili cluster (von Ossowski et al., 2010) and the adhesin encoding *LGG_RS13990* gene. Further research is necessary to investigate if defects in these two minor adhesive units of *L. rhamnosus* GG have an impact on the overall adhesion capacity of the *L. rhamnosus* GG via changes in their own adhesive function or by affecting the SpaCBA pili.

Another mutated gene that might affect pilin production in the Class II derivative PS24, could be the *farR* transcriptional regulator belonging to the gluconate operon repressor (GntR) family (*LGG_RS13185, LGG_02757*). A member of this family, YtrA, has been shown to regulate expression of type III protein secretion system and control several biological processes, such as adhesion and extracellular enzyme production, in a plant pathogen *Xanthomonas citri* (Zhou et al., 2017). Further of note, are the SNPs in several metal and cation transporters: *LGG_RS11535* (*LGG_02411, mntH*), *LGG_RS11610* (*LGG_02426, psaA*), *LGG_RS12705* (*LGG_02658*) and *LGG_RS11565* (*LGG_02417*). ABC transporters are a group of enzymes coupling substrate transport to hydrolysis of ATP. In *Streptococcus pneumoniae*, mutations in PsaA, characterized as a component of a Mn^2+^-transporter, have been shown to cause deficiencies in virulence and adherence of the bacterium (Johnston et al., 2004). Several studies have addressed the connections between the function of certain metal ABC transporters and secretion of fimbrial proteins (Fenno et al., 1995; Claverys, 2001). These data suggested the coupling of pilin secretion and metal ion transport. Additionally, metal ions may act as co-factors for many proteins: manganese has been shown to be important for the function of fibronectin binding protein (Fbp68) of *Clostridium difficile* (Lin et al., 2011). These findings in other strains, coupled to our observations might indicate that cation metabolism plays a role in steering pilin secretion. The potential impact of cation metabolism was confirmed in transcriptomic evaluation of the PS24 derivative, with a whole cluster of genes linked to metal ion transport and regulation being among the top differentially expressed genes compared to wild-type *L. rhamnosus* GG (Figures 2A,C). Here again the overexpression of the *mntH* managanese transporter stands out, the overproduction of which has recently been linked to increased antifungal activity (Siedler et al., 2020).

This transcriptomic analysis also confirmed the increase in transcription of the *spCBA-srtC1* gene cluster in the PS24 strain (Figures 2A,B), which is in accordance with our observations of a higher degree of piliation in Class II strains (Figure 1). The higher piliation phenotype, especially the increase of SpaC at the strains surface, is likely caused by more efficient gene transcription of the pilins. Indeed, especially the transcription counts of the *spaC* gene are strongly increased in an absolute sense compared to the other genes of the *spaCBA-srtC1* operon in PS24, both at OD_600 *nm*_ 2 and OD_600 *nm*_ 7 (Supplementary File S4). A potential explanation is that one of the other genes that have been affected by the EMS mutagenesis normally acts as a repressor of the *spaCBA* promoter that is mapped in the IS30 element upstream of the *spaC* gene (Douillard et al., 2013). Our results indicate that genes related to metal cation homeostasis potentially play a role in regulating *L. rhamnosus* GG adhesion properties, with mutations in key regulators and transporters potentially affecting pilin secretion causing the observed phenotype. Furthermore, this effect seems to be situated at the transcription level. Further research is needed to address the details of the transcriptional regulation of the *spaCBA* gene cluster identified here.

Similar efforts, i.e., using single-molecule genomic analysis to identify SNPs in genes potentially affecting SpaCBA pili, were performed for another Class II derivative, PS31 (Supplementary File S2). This, however, did not lead to clear hypotheses on genes that might drive its highly piliated phenotype, illustrating that much remains to be uncovered on the regulation of SpaCBA biogenesis and functionality in *L. rhamnosus* GG. One lead might be the SNP in the transcriptional regulator belonging to the MarR family (*LGG_RS10240*, *LGG_02127*). A regulator in *Enterococcus faecalis* belonging to the same family, SlyA, has been shown to be involved in virulence and persistence in the host, and similar effects have been noted for other MarR regulators in other pathogens (Michaux et al., 2011). Additionally, Obg, a CgtA GTPase, plays a role in the bet-hedging behavior of *Escherichia coli* and *Pseudomonas aeruginosa*, increasing their persistence and resistance toward stresses (Verstraeten et al., 2015). It remains to be confirmed if and how these genes play a role in SpaCBA pilin regulation and function in *L. rhamnosus* GG.

Taken together, we report on the generation and characterization of highly adherent derivatives of the model probiotic *L. rhamnosus* GG. Given its strong mucus adhesion properties attributed to its unique SpaCBA pili, we investigated if an increase in anti-SpaC IgG interaction translated in an increase in mucus adhesion. According to our phenotypic analyses, we conclude that the observed highly adherent phenotypes of the derivatives was caused either by mutations resulting in a higher adherence ability (Class I) or production of higher quantity of pilins (Classes II–III). We uncovered novel insights in mechanisms regulating the biogenesis and functionality of the SpaCBA pili and propose a coupling between ion homeostasis and piliation in *L. rhamnosus* GG. It will be interesting to investigate and validate these leads further using site-directed mutagenesis approaches in future studies. Our study also delivers a unique set of strains to study the effect of increased quantity of pili on host interactions in human trials, as the strains are not considered as GMOs.

Together with the non-piliated derivatives of *L. rhamnosus* GG (Rasinkangas et al., 2014) we characterized earlier, the here described highly adherent derivatives make up a complete toolset of strains that can aid in further functional dissection of *L. rhamnosus* GG – host interactions, and particularly the role of the SpaCBA pili therein. These insights will ultimately stimulate the development of next-generation probiotics and identification of probiotic effectors.

## Data Availability Statement

The datasets presented in this study can be found in online repositories and Supplementary Material. The names of the repository/repositories and accession number(s) can be found in the article.

## Author Contributions

PR, FD, AP, and WV contributed to conception and design of the study. PR and HT carried out the experimental procedures. JRe performed the immuno EM analysis. JRi was responsible for Illumina sequence processing of all strains. SS provided the human mucus and provided the general comments. HT and PR wrote the manuscript and made the figures. All the authors contributed to manuscript revision, read, and approved the submitted version.

## Conflict of Interest

The authors declare that the research was conducted in the absence of any commercial or financial relationships that could be construed as a potential conflict of interest.

## Supplementary Material

FILE S1 SNPs identified by shotgun Illumina sequencing in all highly adherent derivatives. Includes an SNP comparison sheet where similar SNPs in genes and intergenic regions between all selected derivatives are highlighted: green for the ones shared amongst all highly adherent derivatives and blue for the SNPs common amongst all Class I strains.

FILE S2 SNPs identified by single-molecule genome PacBio resequencing in PS17, PS24, PS31, and PS41.

FILE S3 Whole Genome Alignment results for PS24 and PS31 resulting from PacBio sequencing.

FILE S4 RNASeq count tables of *L. rhamnosus* GG, PB12 and PS24 and differential expression of *L. rhamnosus* GG vs. PS24 at OD2 and OD7.
