# Supplementary material for: Characterization of Highly Mucus-Adherent Non-GMO Derivatives of Lacticaseibacillus rhamnosus GG
Source: Front Bioeng Biotechnol. 2020 Aug 19;8:1024. doi: 10.3389/fbioe.2020.01024 (PMC7466733; doi:10.3389/fbioe.2020.01024)
Supplement: FILE S3 — Whole Genome Alignment results for PS24 and PS31 resulting from PacBio sequencing. [file Table_3.DOCX]

**PacBio Whole Genome Alignment Results**

**Quality statistics of Pacbio sequence reads**

**
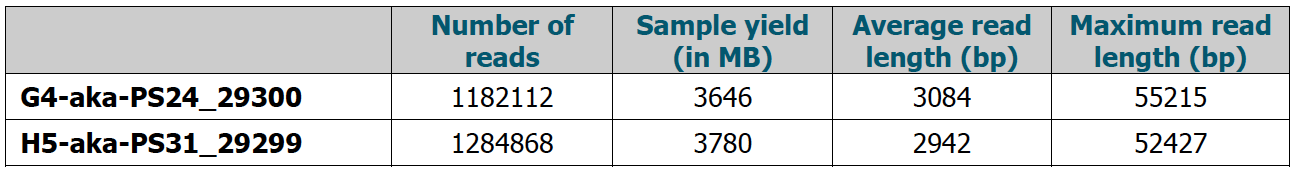
**

***De novo* assembly statistics**

**
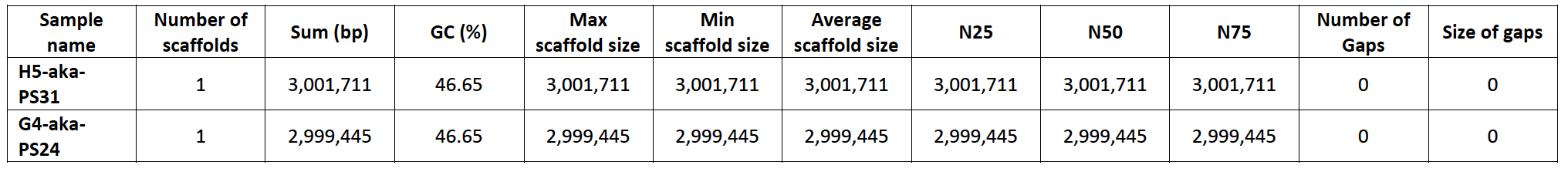
**

**Dot plot of whole genome alignment of *L. rhamnosus* GG vs. PS24**

**
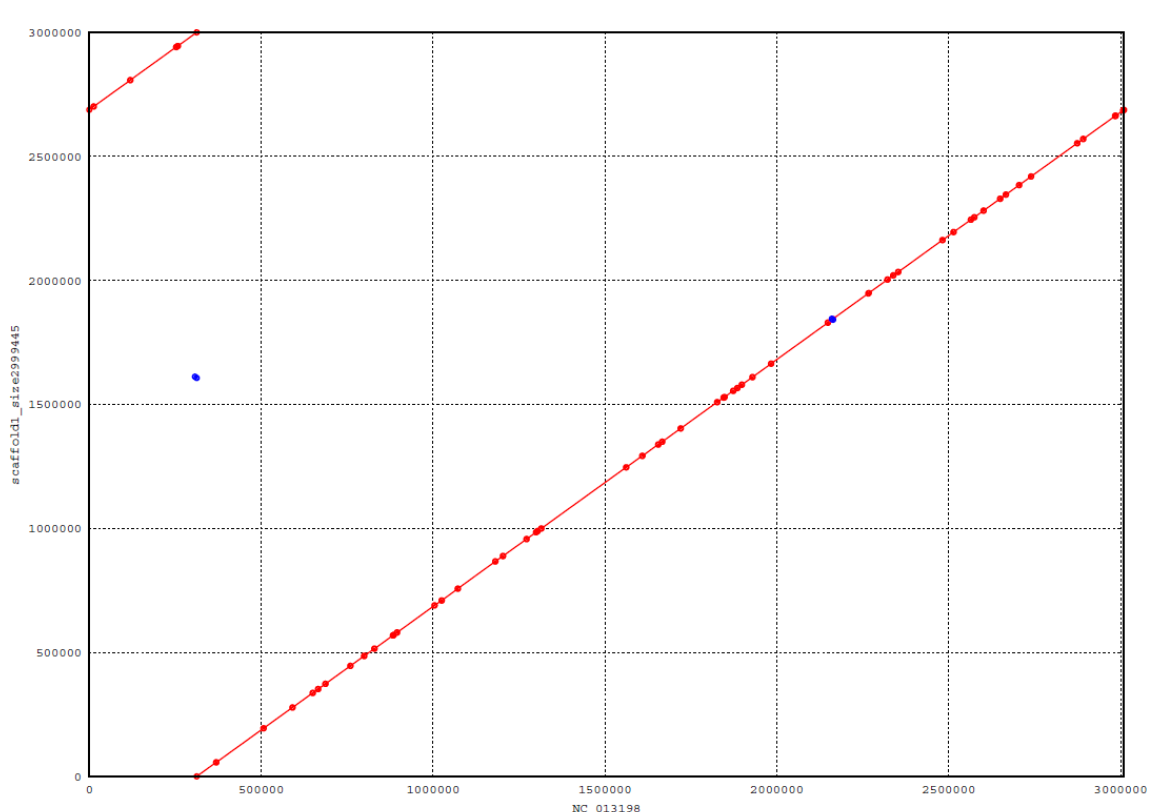
**

**Dot plot of whole genome alignment of *L. rhamnosus* GG vs. PS31**

**
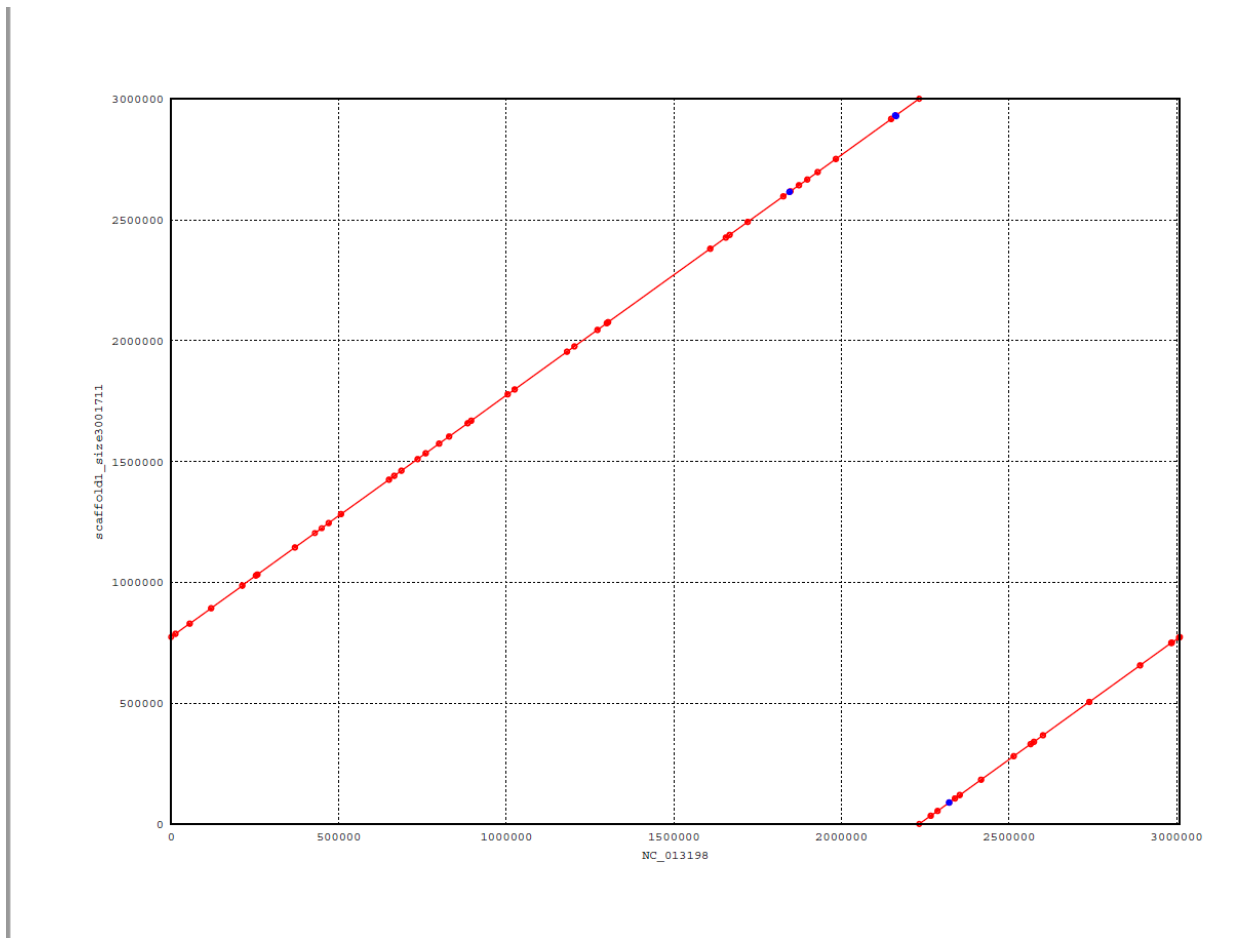
**
